# Characteristics of sagittal spinopelvic alignment in patients with Parkinson’s disease presenting with dropped head syndrome: a case series study

**DOI:** 10.1186/s12883-023-03205-7

**Published:** 2023-04-27

**Authors:** Hiroo Terashi, Kenji Endo, Hitoshi Aizawa

**Affiliations:** 1 Department of Neurology, Tokyo Medical University, 6-7-1 Nishishinjuku, Shinjuku-ku, Tokyo, 160-0023 Japan; 2 Department of Orthopedic Surgery, Tokyo Medical University, 6-7-1 Nishishinjuku, Shinjuku-ku, Tokyo, 160-0023 Japan

**Keywords:** Parkinson’s disease, Dropped head syndrome, Sagittal spinopelvic alignment, Postural deformity

## Abstract

**Background:**

Dropped head syndrome (DHS) is a rare specific abnormal posture known to develop in Parkinson’s disease (PD). This case series study aimed to characterize DHS by analyzing the characteristics of sagittal spinopelvic alignment in patients with PD/DHS.

**Methods:**

The study included eight patients with PD/DHS (men = 3, women = 5; mean age, 68.1 ± 6.4 years). Sagittal spinopelvic alignment was evaluated using 10 parameters on whole-spine lateral radiographs.

**Results:**

The time from the onset of PD to that of DHS varied among the patients from 0 to 15.3 years. In three patients, DHS appeared before the diagnosis of PD. The severity of motor symptoms at DHS onset varied from modified Hoehn and Yahr stage 1 to 4 among the patients. Although the spinopelvic parameters differed among PD/DHS individuals, all patients exhibited cervical kyphosis (cervical lordosis < 0˚). In patients with a larger T1 slope and greater thoracic kyphosis, anterocollis tended to be more severe. According to the assessment of the sagittal vertical axis (SVA), half of the patients showed a positive SVA (SVA ≥ 0 mm), whereas the other half showed a negative SVA (SVA < 0 mm).

**Conclusion:**

DHS appeared regardless of the duration or severity of PD. Although all patients with PD/DHS exhibited cervical kyphosis, the C7 plumb line was shifted anteriorly in half of the patients and posteriorly in the other half.

## Introduction

Parkinson’s disease (PD) is a neurodegenerative disease that mainly affects middle-aged and elderly people and has increasing incidence rates seen in many countries, including Japan [1]. PD has characteristic motor features, such as bradykinesia, resting tremor, rigidity, and abnormal posture [2]. Although the typical abnormal posture in PD is a stooped appearance with flexion of the hips and knees and rounding of the shoulders, some patients may exhibit more severe abnormal posture, such as kyphoscoliosis, camptocormia, Pisa syndrome, or dropped head syndrome (DHS) [2, 3]. Camptocormia is a postural disorder in the sagittal plane characterized by marked flexion of the thoracolumbar spine [2]. DHS is known as anterocollis, with the forward neck flexion exaggerated compared to truncal postural changes [2]. DHS is considered a common feature in multiple system atrophy; however, in patients with PD, DHS is relatively rare, with a reported incidence (in PD) of approximately 6% [2]. To our knowledge, only a few reports have described DHS in PD, and details of its pathology remain to be elucidated. This study aimed to clarify the characteristics of DHS based on sagittal spinopelvic alignment in patients with PD and DHS.

## Materials and methods

### Patients

This case series study examined eight patients (three men and five women; mean age, 68.1 ± 6.4 years) with idiopathic PD who presented with DHS at the Outpatient Clinic of the Department of Neurology, Tokyo Medical University Hospital, between October 2018 and March 2021 (Table 1). In the present study, DHS was defined as when (1) the patient could not maintain a neutral cervical position and gradually developed a chin-on-chest position, and (2) cervical deformity was correctable in supine position [4]. The inclusion criteria were as follows: (1) age ≤ 80 years, (2) modified Hoehn and Yahr (HY) stage ≤ 4, and (3) availability of whole-spine lateral radiographs. We also applied the following exclusion criteria in patient selection: (1) presence of other neurodegenerative diseases; (2) history of spinal, hip, or knee surgery; (3) history of stroke; (4) presence of normal pressure hydrocephalus; and (5) lack of patient interest in voluntary study participation. The diagnosis of PD was based on the United Kingdom Parkinson’s Disease Society Brain Bank criteria [5]. At our hospital, we recommend that all consenting PD patients with abnormal posture undergo periodic spine radiography (approximately every 2 years).

**Table 1:** Clinical features of patients with Parkinson’s disease and dropped head syndrome

Case	Age (years)	Sex	BMI (kg/m^2^)	Time since onset of motor symptoms (years)	modified HY stage	DHS		LEDD (mg)	DADD (mg)
						onset	Adjustment of antiparkinsonian drugs		
1	60	F	22	2.8	1	subacute	improvement	188	188
2	77	F	19	3.6	2	subacute	no improvement	300	0
3	71	M	21	6.1	2	subacute	no improvement	700	200
4	70	M	22	8.5	3	subacute	no improvement	525	225
5	74	M	23	15.3	4	subacute	no improvement	1368	120
6	59	F	30	0	2.5	chronic	no improvement	0	0
7	65	F	23	0.3	4	chronic	improvement	0	0
8	69	F	18	2.4	2	chronic	no improvement	0	0

BMI, body mass index; DHS, dropped head syndrome; modified HY stage, modified Hoehn and Yahr stage; LEDD, L-dopa equivalent daily dose; DADD, dopamine agonist daily dose

This study was approved by the Tokyo Medical University Medical Ethics Committee (#T2020-0110) and was conducted in accordance with principles of the Declaration of Helsinki [6]. Prior to their inclusion in the study, all participants signed written informed consent.

### Measurement and assessment of spinopelvic alignment

**Fig. 1:**
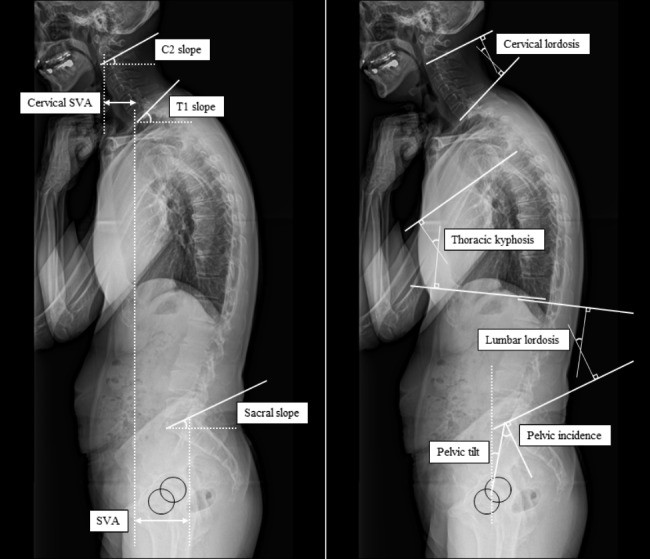
Radiographic parameters of sagittal alignment. (SVA, sagittal vertical axis)

Spinopelvic alignment was assessed by a single examiner using a single whole-spine lateral radiograph. Patients with motor fluctuations underwent the examination during the “on” phase. Before imaging, the patient was instructed to relax while assuming the posture, as directed. Following a standardized protocol, lateral standing radiographs were obtained using vertical films and a radio-opaque calibration tool, with a constant distance between the patient and the radiation source; the patient stood in a fist-on-clavicle position and was instructed to look straight ahead with their knees locked [7, 8]. The following spine parameters were assessed: (1) the sagittal vertical axis (SVA; the distance between the C7 plumb line and the S1 superio-posterior corner), interpreted as global spinal alignment; (2) cervical SVA (the distance between the plumb line from the center of the C2 vertebral body and the posterior superior corner of C7), C2 slope (the angle between the horizontal plane and C2 inferior endplate), and cervical lordosis (the lordotic angle between the C2 and C7 inferior endplates), interpreted as cervical alignment; (3) T1 slope (the angle between the horizontal plane and T1 superior endplate) and thoracic kyphosis (the kyphotic angle between the T5 superior endplate and T12 inferior endplate), interpreted as thoracic alignment; (4) lumbar lordosis (the lordotic angle between the L1 superior endplate and sacral plate), interpreted as lumbar alignment; (5) sacral slope (the angle between the horizontal plane and sacral plate), pelvic tilt (the angle between the line connecting the midpoint of the sacral plate to the bicoxofemoral axis and the vertical line from the bicoxofemoral axis), and pelvic incidence (the angle between the line perpendicular to sacral plate and the line connecting the midpoint of the sacral plate to the bicoxofemoral axis), interpreted as pelvic alignment [8–11] (Fig. 1). Pelvic incidence represented the sum of the sacral slope and pelvic tilt. These are positional and related to pelvic orientation [10]. In reference to the previous classification of cervical alignment by SVA [4, 12], we divided patients with DHS into two groups according to SVA: positive balanced DHS (SVA ≥ 0 mm) and negative balanced DHS (SVA < 0 mm).

### Clinical assessment

The DHS onset pattern was classified as subacute onset (defined as a period of ≤ 2 weeks between the onset of neck symptoms, including posterior cervical pain, and the diagnosis of DHS), and chronic onset (defined as a period of ≥ 2 weeks between the onset of neck symptoms and diagnosis of DHS). Motor symptoms were evaluated using the modified HY stage. Antiparkinsonian medications used by the patients at the time of the DHS onset are presented as their L-dopa equivalent daily doses (LEDD) [13]. In addition, dopamine agonists (DA) are presented as their dopamine agonist daily doses (DADD).

## Results

### Clinical characteristics of patients with PD and DHS

The period between PD appearance and DHS onset varied from 0 to 15.3 years among the patients. In three patients, DHS developed before the diagnosis of PD and ^123^I-metaiodobenzylguanidine myocardial scintigraphy showed a decreased heart-to-mediastinum ratio, a characteristic feature of PD. The severity of motor symptoms at DHS onset varied from modified HY stage 1 to 4 among the patients. With regard to the causal relationship between DHS onset and drug use, DA (pramipexole) was considered to be associated with DHS onset in one patient (Case 1), whereas no apparent association was reported in all the other patients. With regard to the patterns of DHS onset, a subacute pattern was noted in five DHS patients and a chronic pattern was observed in the three patients diagnosed with DHS before PD (Cases 6, 7, and 8). All patients had stiffness in the neck extensor muscle. Although modifications to the dose/type of antiparkinsonian medications improved DHS in two patients (reduction of DA doses in Case 1 and administration of L-dopa in Case 7), no apparent improvement was observed in the remaining six patients (Table 1).

### Characteristics of sagittal spinopelvic alignment

**Fig. 2:**
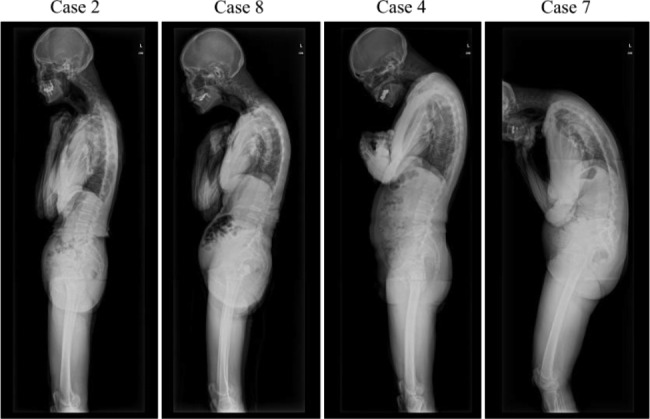
Sagittal spinopelvic alignment in patients with Parkinson’s disease presenting with dropped head syndrome. All patients exhibited cervical kyphosis. As the T1 slope and thoracic kyphosis increased, anterocollis tended to be more severe

Visual assessment of spinopelvic alignment in patients with PD/DHS showed that they characteristically exhibited kyphosis from the cervical to the upper thoracic spine, but the severity varied (Fig. 2). With regard to the spinopelvic parameters, all patients with PD/DHS exhibited cervical kyphosis (cervical lordosis < 0˚). In patients with a larger T1 slope and greater thoracic kyphosis, anterocollis tended to be more severe (Table 2; Fig. 2). According to the assessment of the SVA, half of the patients (Cases 3, 5, 7, and 8) showed positive balanced DHS (SVA ≥ 0 mm), whereas the other half (Cases 1, 2, 4, and 6) showed negative balanced DHS (SVA < 0 mm) (Table 2).

**Table 2:** Spinopelvic alignment among patients with Parkinson’s disease and dropped head syndrome

Case	SVA (mm)	Cervical SVA (mm)	C2 slope (˚)	Cervical lordosis (˚)	T1 slope (˚)	Thoracic kyphosis (˚)	Lumbar lordosis (˚)	Pelvic tilt (˚)	Pelvic incidence (˚)	Sacral slope (˚)
1	-10	47	67	-54	10	24	66	16	68	52
2	-20	57	59	-37	20	17	31	22	55	33
3	18	89	93	-51	40	39	52	16	54	38
4	-13	72	89	-43	50	35	50	18	51	33
5	32	68	51	-11	40	41	31	25	52	27
6	-64	63	103	-81	22	22	44	21	51	30
7	193	83	114	-45	62	42	15	49	47	-2
8	9	74	75	-42	38	39	19	34	54	20

SVA, sagittal vertical axis

## Discussion

In the present study, 50% (4/8) of the patients developed DHS within three years of PD onset, including the three who developed DHS before the diagnosis of PD. In a study of 15 patients with PD and DHS, Kashihara et al. [14] reported the development of DHS within three years of PD onset in seven patients, including one who developed DHS before the onset of parkinsonism. In the Movement Disorder Society Clinical Diagnostic Criteria for PD, “disproportionate anterocollis (dystonic) within the first 10 years” is considered to be a red flag [15]. The current study indicated that DHS could appear in the early stage of PD, suggesting that DHS should be considered in the diagnosis of PD. It was also suggested that the differential diagnosis of neurodegenerative diseases, such as PD, is crucial for the management of patients with DHS.

To our knowledge, this study is the first to report on the characteristics of spinopelvic alignment in patients with PD/DHS. In a Japanese cohort survey on sagittal spinal alignment in the general elderly population [9], the SVA values of healthy individuals in their 60 and 70s were 9 ± 38 and 22 ± 30 mm in men, and 5 ± 30 mm and 30 ± 36 mm in women, respectively. Furthermore, the respective cervical SVA values in the same age groups were 28 ± 8 and 29 ± 12 mm in men and 16 ± 8 and 17 ± 11 mm in women, respectively [9]. In the present study, the cervical SVA values in patients with PD/DHS tended to be greater when compared to the data on the general elderly population. However, the SVA values differed among patients and exhibited no specific trend. A recent study reported that patients with cervical kyphotic deformity exhibit two different types of thoracolumbar compensatory mechanisms according to the C7 plumb line: the head-balanced and trunk-balanced types [12]. In the former type, the center of gravity of the head-plumb line is located in the pelvis due to a posterior shift in the C7 plumb line (negative SVA). This type is characterized by a small T1 slope, straightened thoracolumbar junction, and lumbar hyper-lordosis [12]. In contrast, in the trunk-balanced type, the center of gravity of the head-plumb line is located anteriorly due to the absence of a posterior shift in the C7 plumb line (positive SVA) [12]. This type is characterized by a large T1 slope and low lumbar lordosis. A retrospective observational study in Japan reported that the global sagittal alignment parameter including SVA might have notable impacts on the surgical outcomes of DHS [16]. In our study, four patients with PD/DHS showed a posterior shift of the C7 plumb line, and the remaining four patients showed an anterior shift of the C7 plumb line. These results suggest that DHS in PD also exhibits different subtypes with different thoracolumbar compensatory mechanisms according to the underlying pathology.

In a study of patients with DHS without PD or other neurological diseases, Murata et al. [7] classified DHS into the diffuse kyphosis- (cervical lordosis > -10˚) and cervical kyphosis-types (cervical lordosis ≤ -10˚), based on the degree of cervical lordosis. They demonstrated that the T1 slope and T4–T12 thoracic kyphosis were significantly greater in the diffuse kyphosis-type than in the cervical kyphosis-type. In our study, all patients with DHS exhibited cervical kyphosis with severity of ≤ -10˚. However, based on the visual assessment of spinopelvic alignment as well as the assessment of T1 slope and thoracic kyphosis, the severity of kyphosis from the cervicothoracic junction to the thoracic spine also differed among patients with PD/DHS. In patients with a larger T1 slope and greater thoracic kyphosis, anterocollis tended to be more severe.

The pathogenesis of DHS in PD is poorly understood. Fujimoto [17] identified dystonia of the flexor neck muscles and weakness of the extensor neck muscles as the pathophysiological mechanisms of DHS in PD, highlighting the involvement of isolated neck extensor myopathy in the weakness of the extensor neck muscles. Furthermore, Lava et al. [18] reported a case of levodopa-responsive parkinsonism accompanied by anterocollis due to focal neck extensor myopathy. In contrast, Kashihara et al. [14] reported in a study of 15 patients with PD/DHS that although they had no neck muscle weakness, many patients exhibited predominant neck muscle rigidity and characteristic marked contraction of the neck extensor muscles in the upright position. Our study also showed that all the PD/DHS patients had stiffness in the neck extensor muscle.

Various cases of DHS associated with antiparkinsonian medications, particularly DAs, have been reported, and our study also included one case strongly suggestive of such an association [2]. With regard to DAs, several studies have reported differences in the sensitivity of muscles to L-dopa and other DAs; however, how DA affects neck muscles and triggers DHS remains unknown [17].

There are no consistent guidelines for the treatment of DHS associated with PD. In some cases, improvement occurs after adjustment of antiparkinsonian drugs (including dose reduction and discontinuation of dopamine agonists); therefore, dose adjustment should be attempted as a first-line management strategy. In our study, drug adjustment led to improvement in two out of eight patients. The efficacy of botulinum toxin therapy for DHS is inconsistent. There are fewer reports on the efficacy of surgery for DHS in PD, and the long-term prognosis is unclear. In our study, two patients underwent surgery and showed postoperative improvement of DHS. Future large-scale longitudinal studies are needed to determine the optimal treatment of DHS in PD.

Our study has certain limitations. First, because it was a single-center case series study, the sample size was relatively small, similar to previous reports [14]. We suggested the possible diversity of DHS in PD based on an analysis of the characteristics of sagittal spinopelvic alignment; however, we could not perform detailed analysis of the pathology and could not make a firm conclusion. Second, detailed examinations, such as electromyography and muscle biopsy, were not conducted on our patients, which precluded detailed analysis of the pathogenesis and etiology of DHS. To address these issues, longitudinal studies of a large sample size using electromyography or muscle biopsy need to be performed in the future.

## Conclusion

DHS, which is a rare complication of PD, appeared regardless of the duration or severity of the disease. The assessment of sagittal spinopelvic alignment in patients with PD/DHS showed the presence of cervical kyphosis in all patients. However, half of them exhibited positive balanced DHS, and the other half exhibited negative balanced DHS. To elucidate the pathology of DHS in PD, further studies with a larger sample size are warranted.

## Data Availability

The datasets generated and/or analyzed in this study are not publicly available due to privacy and ethical restrictions but are available from the corresponding author upon reasonable request.

## Abbreviations

DA: dopamine agonist
DADD: dopamine agonist daily doses
DHS: dropped head syndrome
HY: Hoehn and Yahr
IQR: interquartile range
LEDD: L-dopa equivalent daily doses
PD: Parkinson’s disease
SD: standard deviation
SVA: sagittal vertical axis

## References

[1] GBD 2016 Neurology Collaborators (2019). Global, regional, and national burden of neurological disorders, 1990–2016: a systematic analysis for the global burden of Disease Study 2016. Lancet Neurol.

[2] Doherty KM, van de Warrenburg BP, Peralta MC, Silveira-Moriyama L, Azulay JP, Gershanik OS (2011). Postural deformities in Parkinson’s disease. Lancet Neurol.

[3] Rabin ML, Earnhardt MC, Patel A, Ganihong I, Kurlan R (2016). Postural, bone, and joint disorders in Parkinson’s disease. Mov Disord Clin Pract.

[4] Endo K, Kudo Y, Suzuki H, Aihara T, Matsuoka Y, Murata K (2019). Overview of dropped head syndrome (combined survey report of three facilities). J Orthop Sci.

[5] Hughes AJ, Daniel SE, Kilford L, Lees AJ (1992). Accuracy of clinical diagnosis of idiopathic Parkinson’s disease: a clinico-pathological study of 100 cases. J Neurol Neurosurg Psychiatry.

[6] World Medical Association (2013). World Medical Association Declaration of Helsinki: ethical principles for medical research involving human subjects. JAMA.

[7] Murata K, Endo K, Aihara T, Suzuki H, Matsuoka Y, Nishimura H (2020). Relationship between cervical and global sagittal balance in patients with dropped head syndrome. Eur Spine J.

[8] Terashi H, Endo K, Kato H, Ido N, Aizawa H (2022). Characteristics of sagittal spinopelvic alignment in patients with Parkinson’s disease. Acta Neurol Scand.

[9] Uehara M, Takahashi J, Ikegami S, Tokida R, Nishimura H, Sakai N, Kato H (2019). Sagittal spinal alignment deviation in the general elderly population: a japanese cohort survey randomly sampled from a basic resident registry. Spine J.

[10] Le Huec JC, Thompson W, Mohsinaly Y, Barrey C, Faundez A (2019). Sagittal balance of the spine. Eur Spine J.

[11] Dagdia L, Kokabu T, Ito M (2018). Classification of adult spinal deformity: review of current concepts and future directions. Spine Surg Relat Res.

[12] Mizutani J, Verma K, Endo K, Ishii K, Abumi K, Yagi M (2018). Global spinal alignment in cervical kyphotic deformity: the importance of head position and thoracolumbar alignment in the compensatory mechanism. Neurosurgery.

[13] Tomlinson CL, Stowe R, Patel S, Rick C, Gray R, Clarke CE (2010). Systematic review of levodopa dose equivalency reporting in Parkinson’s disease. Mov Disord.

[14] Kashihara K, Ohno M, Tomita S (2006). Dropped head syndrome in Parkinson’s disease. Mov Disord.

[15] Postuma RB, Berg D, Stern M, Poewe W, Olanow CW, Oertel W (2015). MDS clinical diagnostic criteria for Parkinson’s disease. Mov Disord.

[16] Kudo Y, Toyone T, Endo K, Matsuoka Y, Okano I, Ishikawa K (2020). Impact of spinopelvic sagittal alignment on the surgical outcomes of dropped head syndrome: a multi-center study. BMC Musculoskelet Disord.

[17] Fujimoto K (2006). Dropped head in Parkinson’s disease. J Neurol.

[18] Lava NS, Factor SA (2001). Focal myopathy as a cause of anterocollis in parkinsonism. Mov Disord.

